# Supplementary material for: The Role of CPNE7 (Copine-7) in Colorectal Cancer Prognosis and Metastasis
Source: Int J Mol Sci. 2023 Nov 24;24(23):16704. doi: 10.3390/ijms242316704 (PMC10706690; doi:10.3390/ijms242316704)
Supplement: Supplementary file 1 [file ijms-24-16704-s001.zip › ijms-2663516-supplementary.pdf]

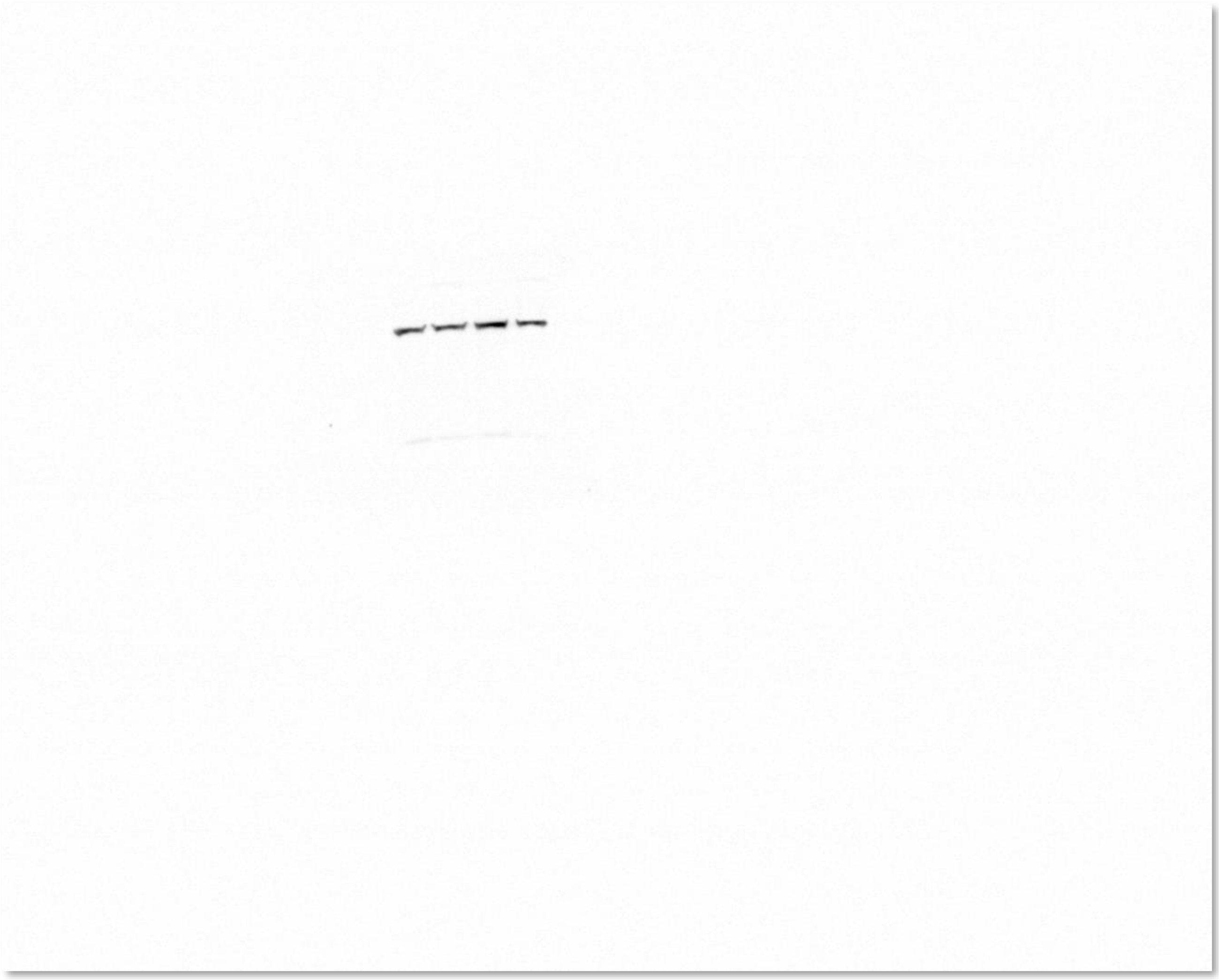

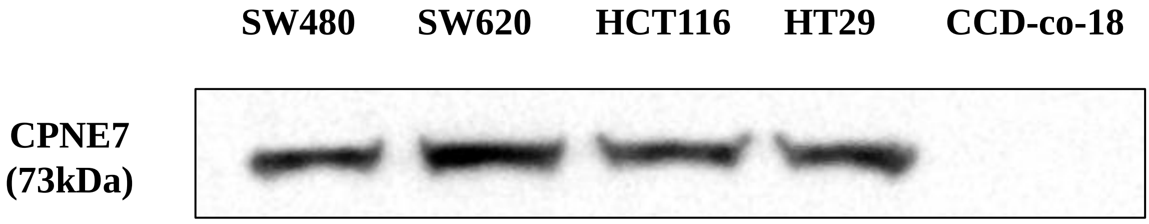

Supplementary Figure S1. Western blot of the fig.1-B.
The *CPNE*7 protein expression level by western blot in five cell lines. (SW480; colon adenocarcinoma, SW620; colorectal carcinoma, HCT116; colorectal carcinoma, HT29; colon adenocarcinoma, DLD1; colon adenocarcinoma, CCD-18-co; colon normal fibroblast.)

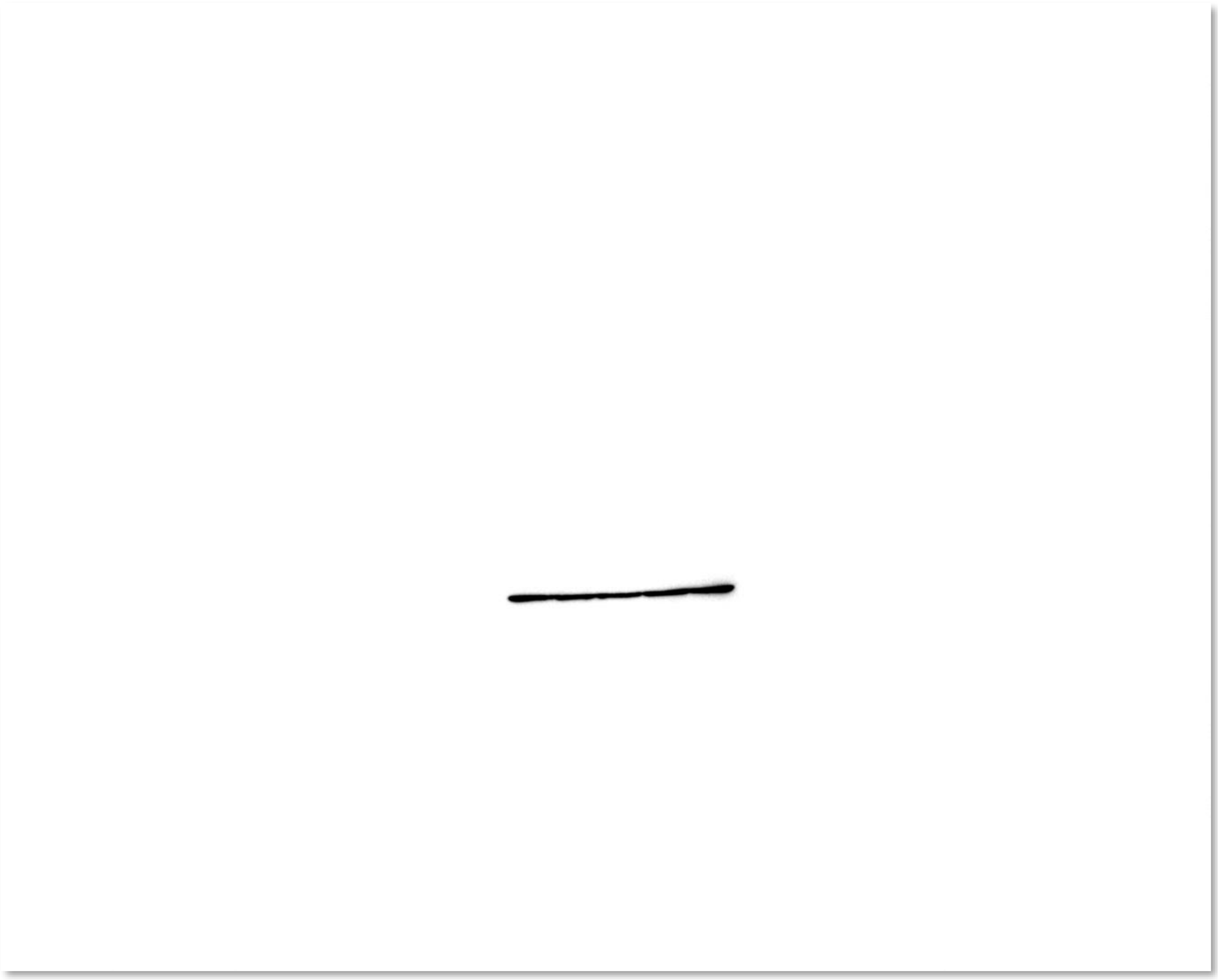

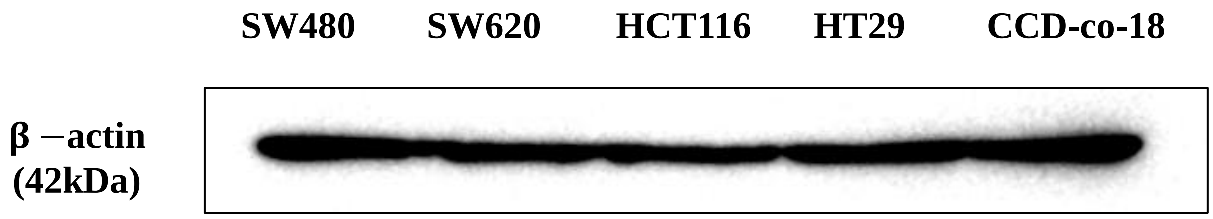

Supplementary Figure S2. Western blot of the fig.1-B.
The *β-actin* protein expression level by western blot in five cell lines .

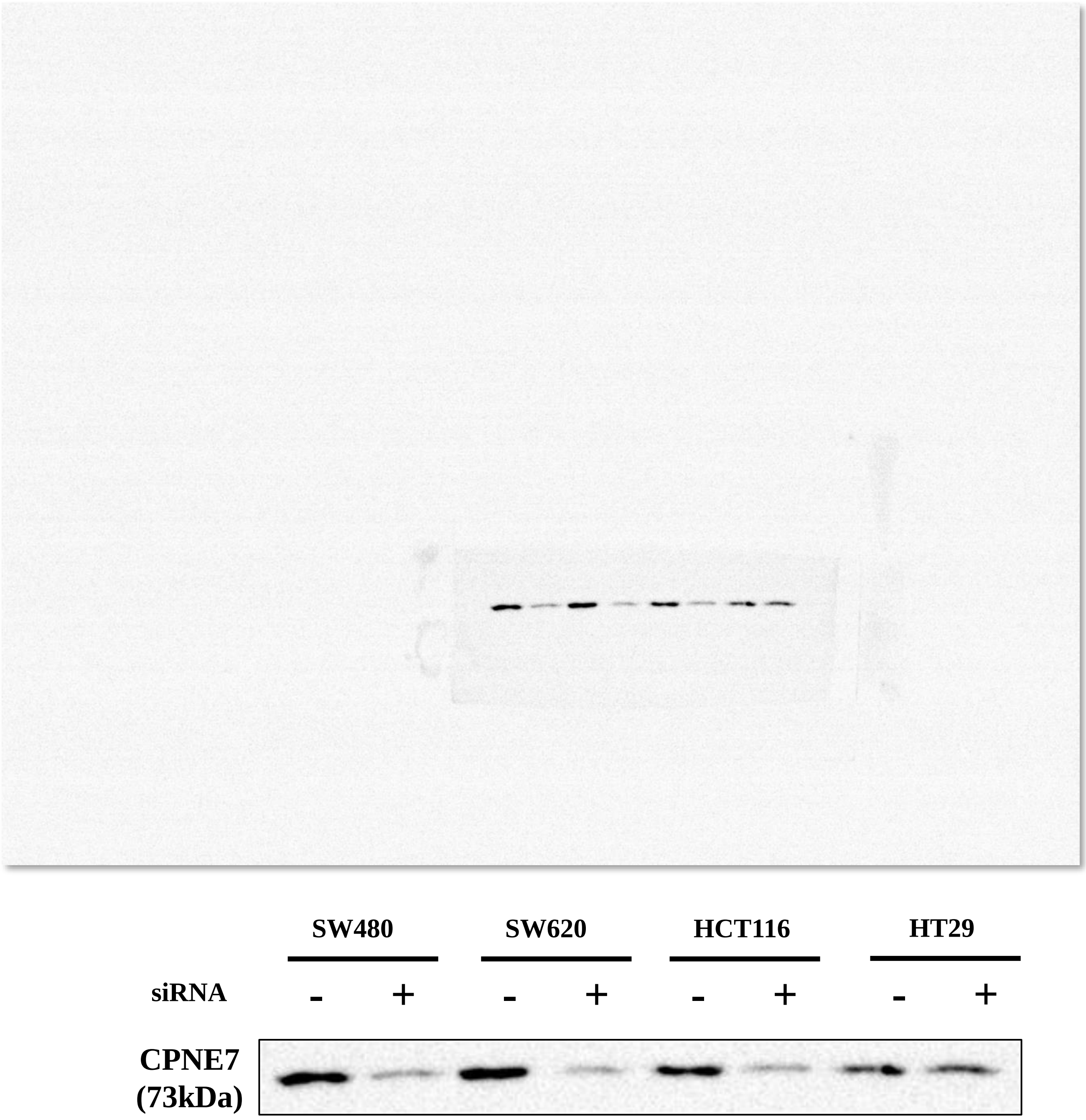

Supplementary Figure S3. Western blot of the fig.2-B. Identify the effects of *CPNE7* siRNA transfection through Western blotting.

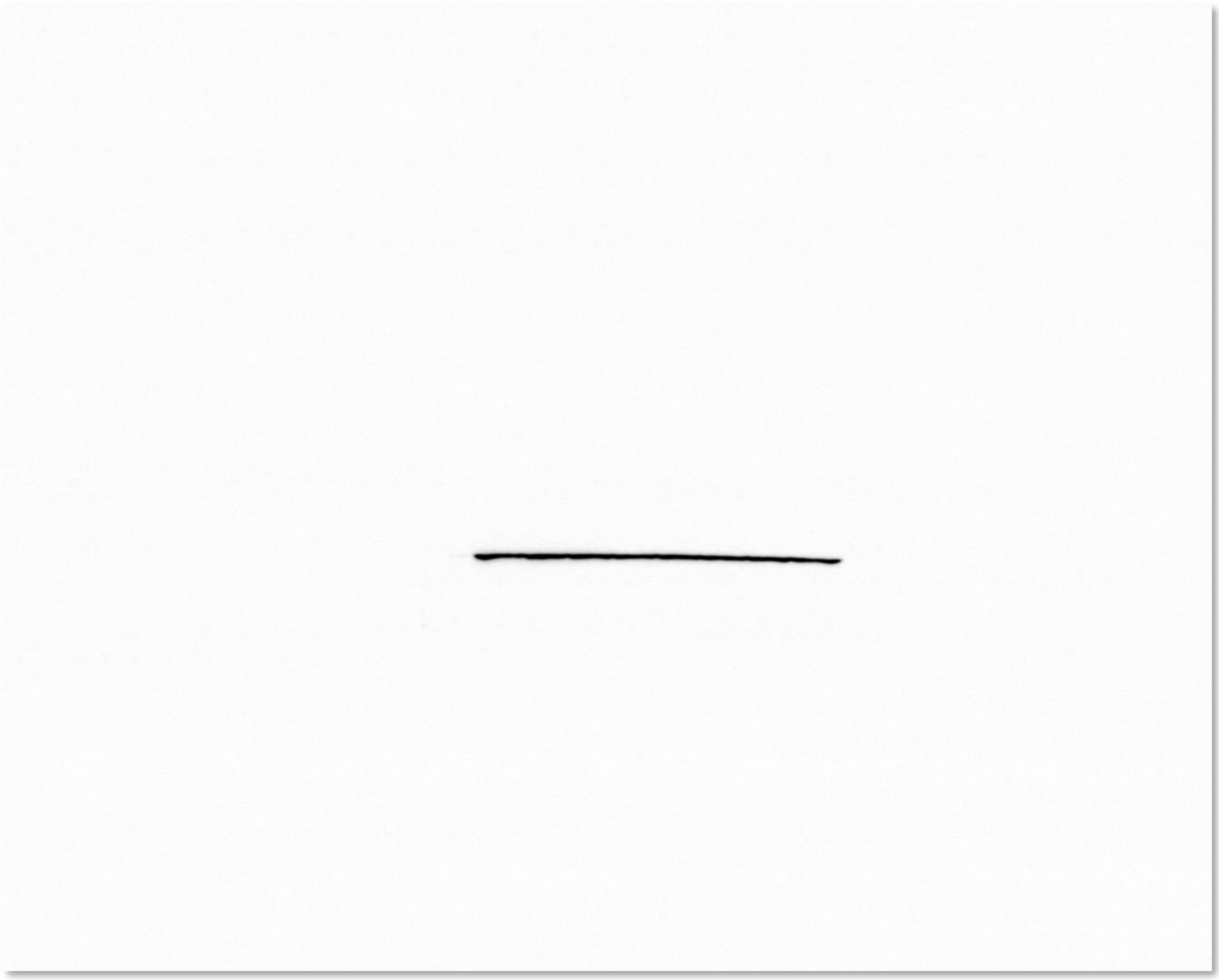

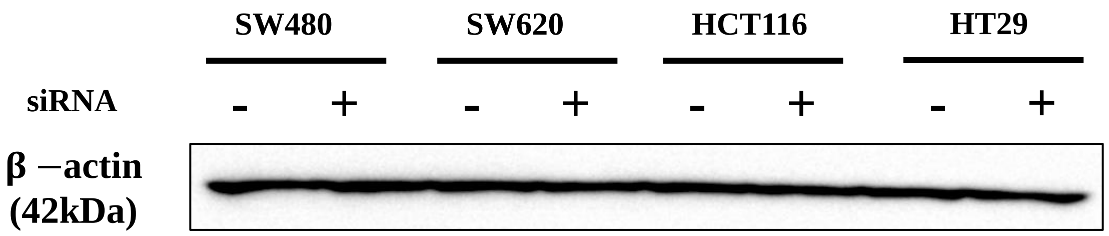

Supplementary Figure S4. Western blot of the fig.2-B. *β-actin* expression was assessed by Western blot in four CRC cell lines and in each cell line infected with *CPNE7* siRNA.

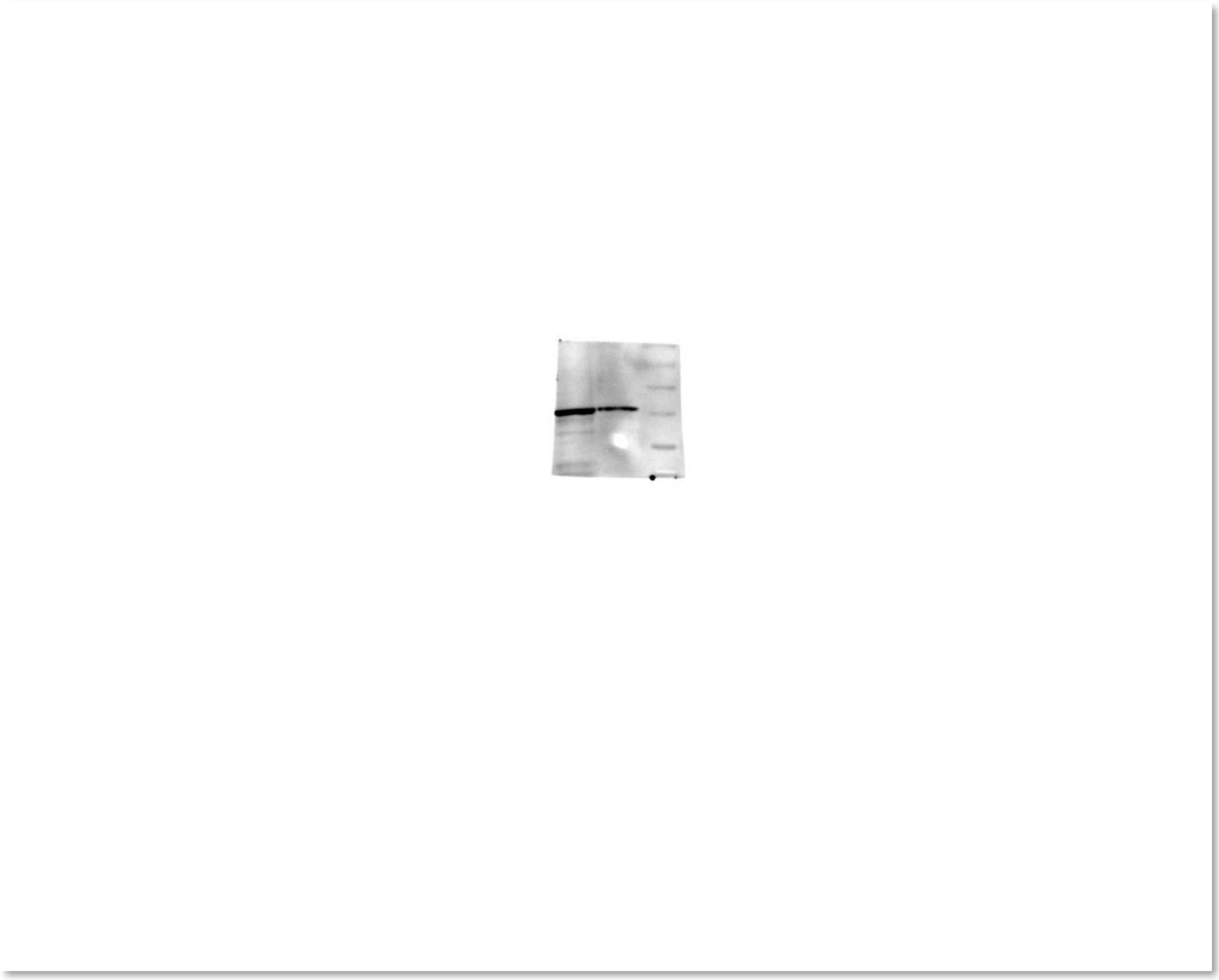

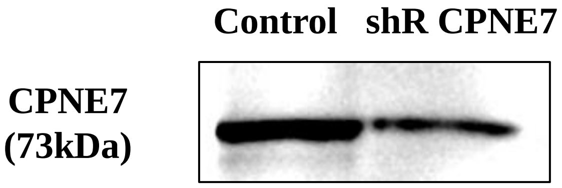

Supplementary Figure S5. Western blot of the fig.3-A. Identify the effects of *CPNE7* siRNA transfection through Western blotting.

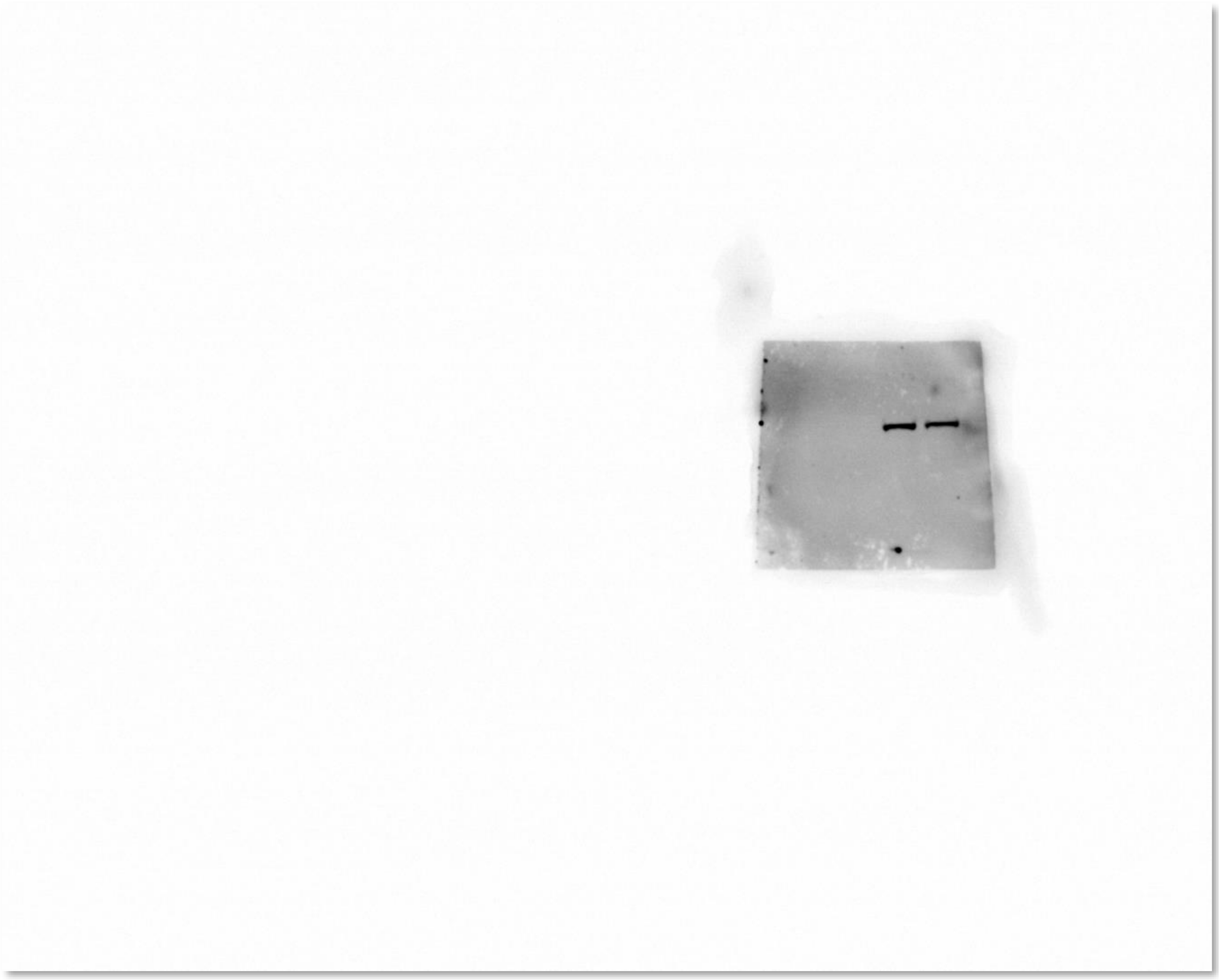

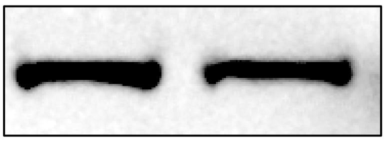

Supplementary Figure S6. Western blot of the fig.3-A.
β-actin expression was assessed by Western blot in SW480 and in cell line infected with *CPNE7* shRNA.
